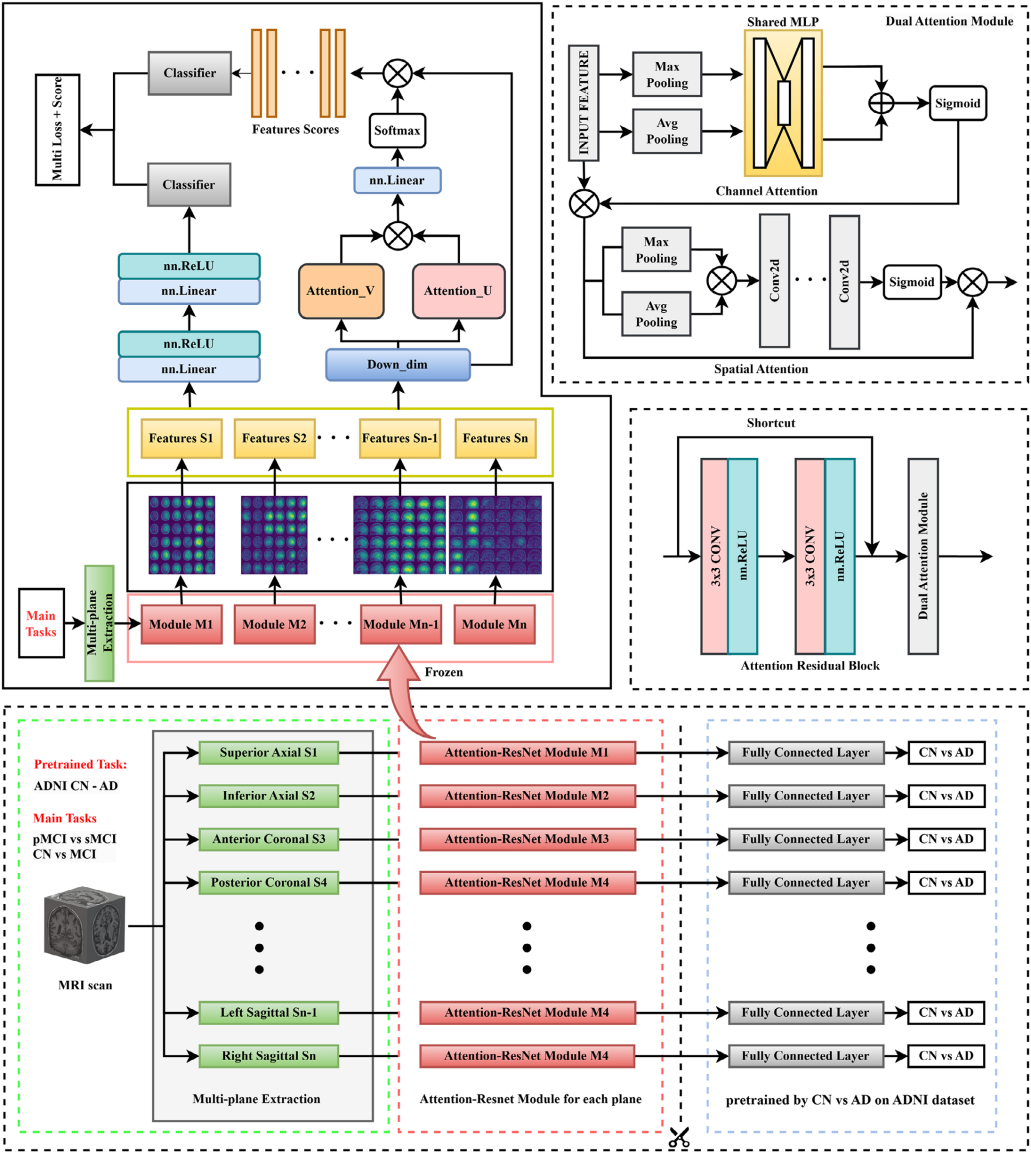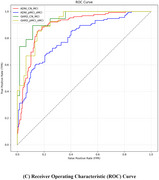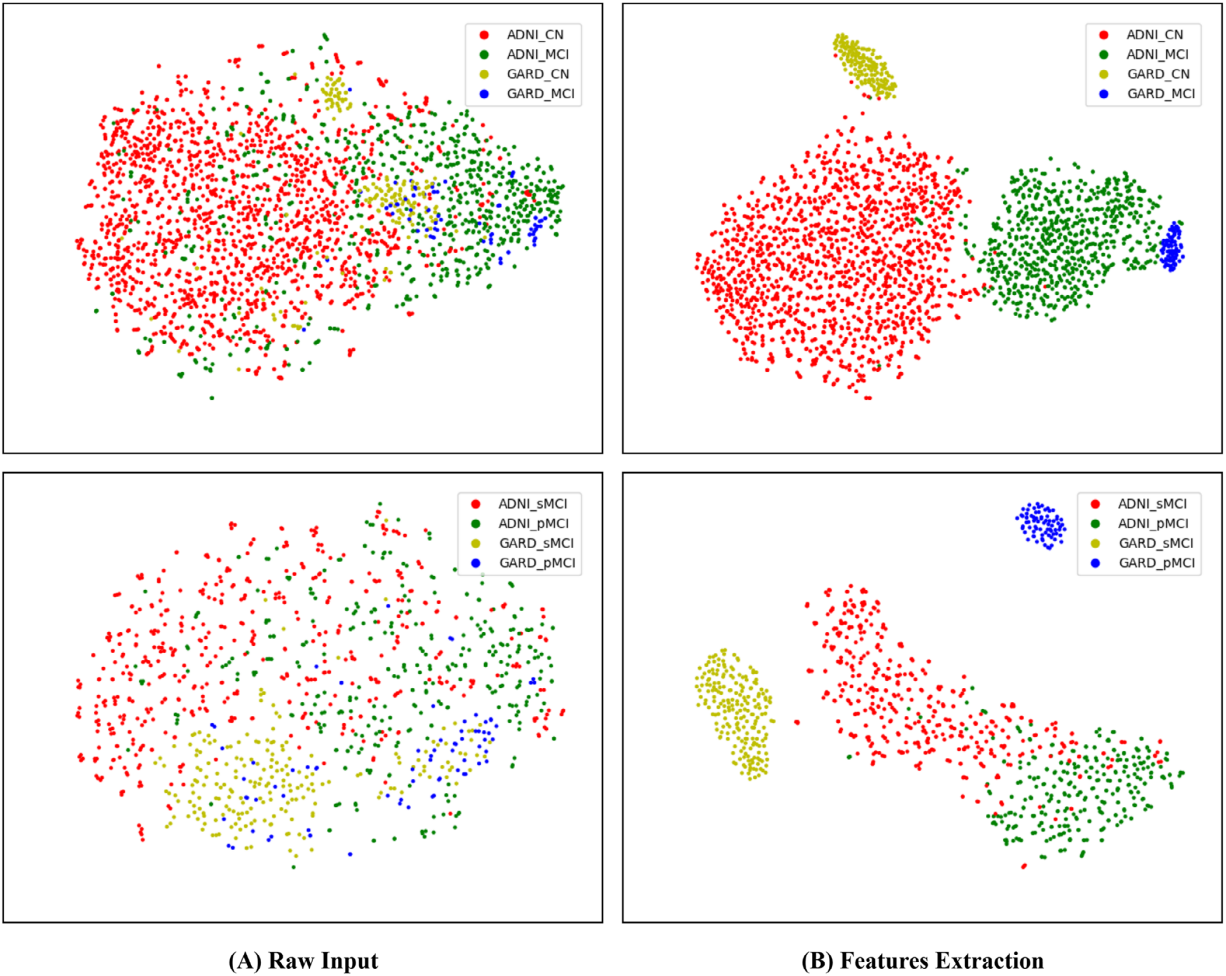# High‐generalizability Deep Learning Framework for Early Detection of Mild Cognitive Impairment Progression to Alzheimer's Disease

**DOI:** 10.1002/alz70856_101596

**Published:** 2025-12-25

**Authors:** Gia Minh Hoang, Jae Gwan Kim

**Affiliations:** ^1^ Gwangju Institute of Science and Technology, Bukgu, Gwangju, Korea, Republic of (South); ^2^ Department of Biomedical Science and Engineering, Gwangju Institute of Science and Technology, Gwangju 61005, Korea, Republic of (South)

## Abstract

**Background:**

Alzheimer's disease (AD) is a progressive neurodegenerative disorder where early diagnosis is critical for effective intervention. Mild Cognitive Impairment (MCI), often considered a prodromal stage of AD, represents a crucial target for early diagnosis. However, predicting which MCI individuals will progress to AD remains a significant challenge due to the heterogeneity of MCI progression. Advanced diagnostic tools are needed to address this challenge. In this study, we propose a high‐generalizability deep learning approach for early diagnosis of MCI progression to AD using Magnetic Resonance Imaging (MRI).

**Method:**

Images were obtained from the Alzheimer's Disease Neuroimaging Initiative (ADNI) and Gwangju Alzheimer's and Related Dementia (GARD) database. Participants were categorized into four groups: Cognitive Normal (CN), MCI, progressive MCI (pMCI), and stable MCI (sMCI). Progressive MCI was defined as individuals who progressed to AD within three years, while stable MCI referred to those who remained in the MCI state during the same period. To address the heterogeneity in MRI data, we employed a multi‐plane feature extraction strategy combined with an attention‐based mechanism, enabling robust classification performance and improved generalizability. The model was trained using the ADNI dataset and validated for generalizability on the independent GARD dataset.

**Result:**

Our approach achieved competitive classification performance on the ADNI dataset, with accuracy and AUC scores of 91.92% and 96.22% for CN vs. MCI, and 78.16% and 83.77% for pMCI vs. sMCI, respectively. In the generalizability evaluation on the GARD dataset, the model maintained robust performance with accuracy scores of 86.25% for CN vs. MCI and 76.14% for pMCI vs. sMCI. t‐SNE visualization confirmed that features extracted by the model were well‐separated across diagnostic groups, demonstrating its discriminative capability.

**Conclusion:**

Early diagnosis of MCI progression to AD is critical for enabling timely interventions that can potentially delay disease progression. Our proposed deep learning framework demonstrated high accuracy and generalizability in diagnosing MCI progression to AD and distinguishing between CN and MCI individuals. These results highlight the potential of our approach as a robust and reliable tool for early diagnosis of Alzheimer's disease, leveraging advanced neuroimaging analysis to support clinical decision‐making.